# Do Individual Effects Reflect Quantitative or Qualitative Differences in Cognition?

**DOI:** 10.5334/joc.171

**Published:** 2021-08-27

**Authors:** Anna-Lena Schubert, Dirk Hagemann, Jan Göttmann

**Affiliations:** 1 Institute of Psychology, University of Mainz, Mainz, Germany; 2 Institute of Psychology, Heidelberg University, Heidelberg, Germany

**Keywords:** individual differences, cognitive tasks, trial numbers

## Abstract

Rouder and Haaf (2020) posed the important question if there are some individuals whose behavior is not in accordance with well-established experimental effects and whether these individual differences are quantitative or qualitative in nature. In our commentary, we discuss the distinction between quantitative and qualitative individual differences and between individual and average causal effects and come to the conclusion that this is not a new question, but in fact one that has already been discussed by Gordon W. Allport (1937) and Donald B. Rubin (1974, 1978). Moreover, we critically examine their proposed rule of thumb to collect about 100 trials per experimental condition to reliably measure individual differences in typical experimental effects. Based on simulation results, we suggest to not rely on any general rule of thumb, but to use simulation studies and the convenient quid function provided by the authors to make more informed decisions regarding trial numbers for specific experimental designs.

Psychological science aims to identify laws that govern human thinking, feeling, and behavior. Unlike the natural sciences, however, we cannot directly measure psychological constructs. Cognitive psychologists study how certain experimental manipulations designed to induce processing demands for these constructs affect performance in an experimental task. Typically, most individuals behave in a manner consistent with well-established experimental effects, while a minority does not show the expected effect or even shows a reverse effect. If this were itself a robust phenomenon – if certain individuals behaved in ways inconsistent with “universal” psychological laws – it would have serious and far-reaching implications for theory building. In physics, for example, a far greater research effort is now devoted to the study of deviations from than confirmations of theoretical predictions. In their paper, Rouder and Haaf (2020) pose the question if these individual deviations from experimental effects are substantive or if they can be accounted for by measurement error. They propose a hierarchical model to distinguish these substantive deviations from measurement error and conclude that there are certain experimental effects from which individuals deviate in a qualitative manner by showing effects in the other direction. We appreciate the elegance of the statistical solution proposed by Rouder and Haaf (2020) and the accessibility of the accompanying R functions. In our commentary, we will focus on two more conceptual parts of their paper: The distinction between quantitative and qualitative individual differences and their proposed rule of thumb for trial number considerations.

## Quantitative vs. qualitative individual differences and individual vs. average experimental effects

The question of quantitative vs. qualitative individual differences has a long tradition in psychology. Already Gordon W. Allport (1937) made a clear distinction between qualitative and quantitative individual differences. He suggested that an “individual trait” is peculiar to a person and as such, has no metric and no population distribution and thus is truly qualitative in nature (c.f. idiographic approach). In contrast, a “common trait” is an aspect of personality that has a metric and that allows a numerical comparison between individuals and thus is quantitative in nature (c.f. nomothetic approach). From this perspective, not much of the present authors’ proposal is “qualitative” because they ground their “qualitative individual differences” on negative values of an effect size measure, which allows a comparison between individuals, has a population distribution, and is therefore quantitative in nature (it corresponds clearly to the common trait in Allport’s terminology).

**Figure 1:**
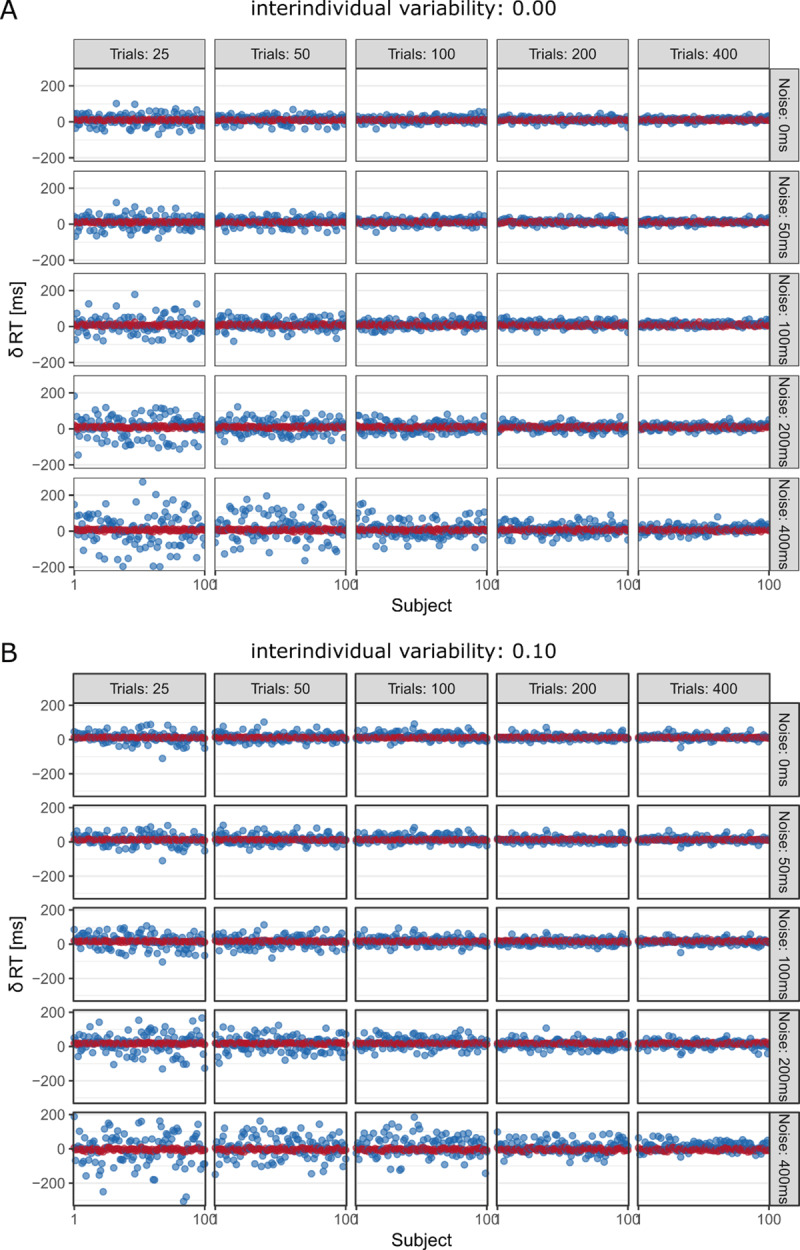
Observed (blue) and model-estimated (red) effects for lower degrees of interindividual variability.

To expand on this issue, the authors did not distinguish between the level of measurement and the level of theoretical interpretation of the measure. The general and individual effect is something that can be measured and is therefore quantitative (elsewise, it would not have been possible to place individuals along a common dimension denoted as “effect” in ***Figure 1***). Perhaps the theoretical interpretation of certain effect size values must shift from one theory to another, depending on the range of effect size values, allowing for a qualitative difference in the interpretation of the values. However, this does not make the values qualitative in nature, they still are quantities. In this context it must be seen that the authors ground their decision about “qualitative individual differences” solely on the outcome of statistical model testing, irrespective of the measured constructs or theoretical considerations. Taking this stance literally, one could measure the temperature in a sample of villages in January and February and find that it increased in most villages but decreased in others. Does this indicate that there are qualitative differences between the villages in terms of theoretical explanation of temperature, i.e. do we need another physics in some of the villages? The answer is certainly no.

We also question that “laterality” is a good example for “obvious qualitative individual differences” (p. 11). Handedness comes in grades, ranging from extreme right-handedness over ambidexterity to extreme left-handedness. As a matter of fact, only few individuals use only one hand exclusively for all one-handed activities (e.g., Springer & Deutsch, 1993). Therefore, handedness questionnaires typically allow to locate a given person on a continuum between extreme left-handedness through equal use of both hands to extreme right-handedness (e.g., Oldfield, 1971). Hence, handedness is not a qualitative but a quantitative trait. The same case can be made for “preferences”, which the authors use as another example for “qualitative” individual differences.

We would also like to highlight that Rubin (1974) published an authoritative paper on the analysis of causal effects, where he made a clear distinction between “individual causal effects” and the “average causal effect”. This paper also provides a discussion of the consequences of the homogeneity vs. heterogeneity of individual causal effects, including outliers, on the interpretation of the average causal effect. He used this distinction between individual and average causal effects for an extended treatment of a Bayesian analysis of randomized studies (Rubin, 1978). Later, Steyer (2005) translated Rubin’s ideas into a contemporary structural equation modeling framework, which allows to estimate the variance as well as the numerical values of individual causal effects (needless to say, random noise is controlled via latent variable modeling). We believe that this work is highly relevant to Rouder’s and Haaf’s (2020) considerations.

In their introduction (p. 2), the authors stated: “Rather than ask an on average question, we advocate a new question: Are there qualitative individual differences?” It is our impression that this question is not new but has been extensively treated in the literature. The target article does not do this previous work justice by ignoring the important milestones made by Alport (1937), Rubin (1974, 1978), and Steyer (2005).

## Trial numbers

Rouder and Haaf (2020) emphasized the need to collect sufficient data to reliably measure interindividual differences in experimental effects. As a rule of thumb, they proposed to collect data from at least 100 trials per experimental condition. We ran a simulation study to illustrate how different factors (number of trials, trial noise, and the degree of interindividual variation) affected the visibility of individual differences. For this purpose, we used the R package *flankr* (Grange, 2016) to simulate behavioral data from the dual-stage two-phase (DSTP) model (Hübner et al., 2010), which is a mathematical model of attention and decision processes in the Eriksen flanker task (Eriksen & Eriksen, 1974). We realized four simulation factors: Number of trials per condition (25, 50, 100, 200 vs. 400), trial noise (0ms, 50ms, 100ms, 200ms, 400ms), and interindividual variability of the stimulus-selection drift rate *v_StimSelection_*, which is the model parameter that describes the speed and efficiency with which individuals focus their attention on the target stimulus (0.00, 0.10, 0.20, .40).

**Figure 2:**
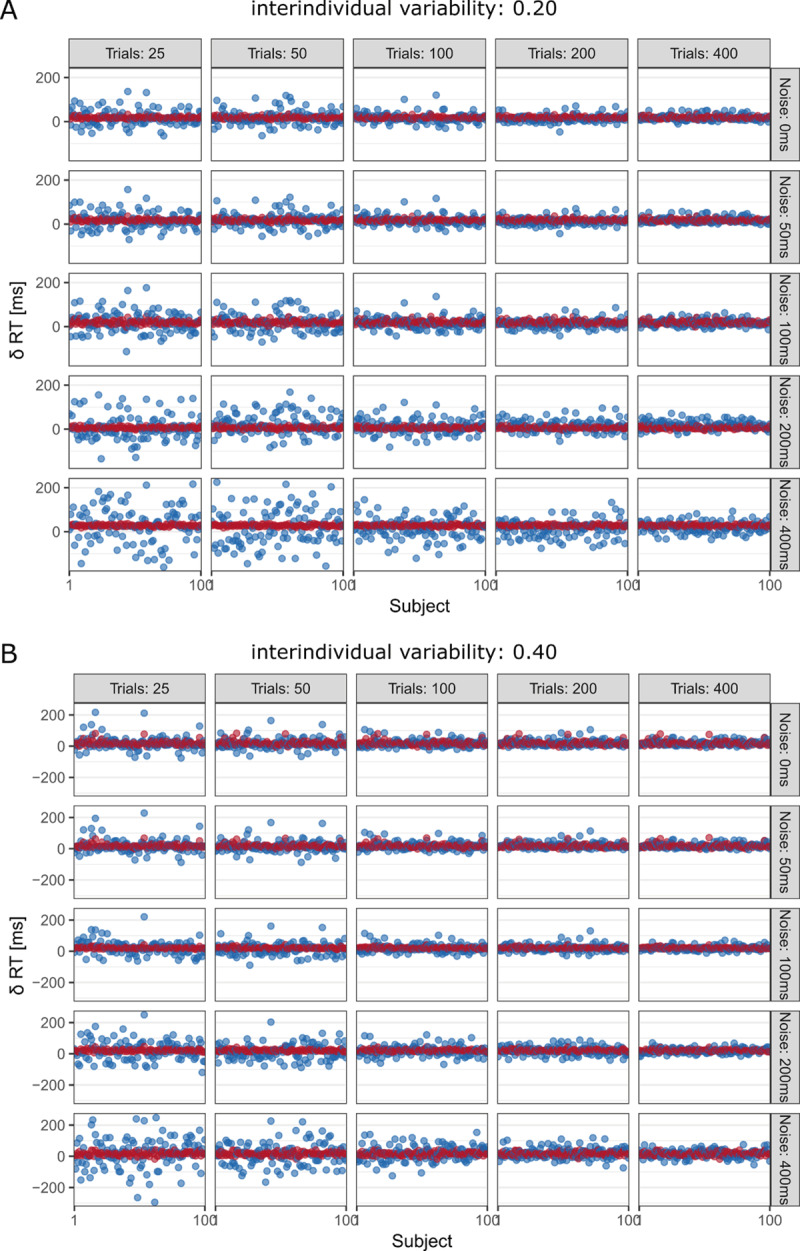
Observed (blue) and model-estimated (red) effects for higher degrees of interindividual variability.

As expected, observed and model-estimated individual experimental effects converged when there was either a high number of trials or a low degree of trial noise (see ***Figures 1*** and ***2***). As soon as there was some true interindividual variation in the latent cognitive process, at least 200 trials per condition were needed to compensate for the effects of moderate-to-high trial noise on the measurement precision of the observed individual experimental effects (see ***Figures 1B*** and ***2***). This result confirms our hunch to not rely on a rule of thumb of collecting at least 100 trials per condition. Instead of replacing it with another arbitrary rule of thumb, we urge to use simulation studies and the convenient *quid* function provided by the authors of the target article to make more informed decisions regarding trial numbers for specific experimental designs whenever possible.
